# The Selection of Storage Period for Fresh Rice Noodles, Processing Materials, and the Screening of Key Indicators

**DOI:** 10.3390/foods13233965

**Published:** 2024-12-09

**Authors:** Zhe Yang, Peng Liu, Xilin Fang, Guanghui Chen

**Affiliations:** 1 Department of Agronomy, College of Agronomy, Hunan Agricultural University, Changsha 410128, China; yangzhe0831@163.com (Z.Y.);; 2 Modern College of Agriculture and Forestry Engineering, Ganzhou Polytechnic, Ganzhou 341008, China; 3 Yuelu Mountain Laboratory of Hunan Province, Hunan Agricultural University, Changsha 410128, China

**Keywords:** paddy storage period, fresh rice noodles’ quality, comprehensive evaluation, rice quality

## Abstract

The storage period of paddy is a critical factor affecting rice quality, and it is still unclear how fresh rice noodles, primarily made from paddy, respond to changes in the storage period. To elucidate the relationship between the paddy storage period and the quality of fresh rice noodles, this study used fourteen rice varieties as materials and set up three paddy storage periods (six months, nine months, and twelve months). It explored the quality variation patterns of fresh rice noodles processed from these paddies and analyzed the relationship between the two in conjunction with rice quality. The results indicated that fresh rice noodles produced from paddies stored for nine months exhibited superior quality compared to the other two storage periods. Grey relational analysis and correlation analysis confirmed that this was primarily attributed to changes in the gel consistency of the paddy. When the paddy was stored for nine months and the rice gel consistency was approximately 32 mm, the quality of the fresh rice noodles produced was optimal, serving as an important basis for selecting raw materials for fresh rice noodles.

## 1. Introduction

Rice noodles are the second most commonly consumed rice product in Asia after rice [1,2] and are highly favored by consumers in China and some Southeast Asian countries (such as Thailand, Sri Lanka, and Cambodia) [3]. They are loved for their rich variety, smooth and refreshing mouthfeel, tender texture, and the versatility to serve both as a staple food and for breakfast [4]. Depending on the moisture content, rice noodles can be categorized into dry rice noodles, semi-dry rice noodles, and fresh rice noodles, with the latter being the mainstream choice in the consumer market due to their fresh taste [5].

Fresh rice noodles are rice products processed through steps such as soaking, grinding, heating gelatinization, and cutting into strips [6]. Factors affecting the quality of fresh rice noodles mainly involve the characteristics of the paddy raw materials, processing techniques, and storage preservation methods, with numerous studies indicating that the characteristics of the paddy raw materials are the most important factors in determining the quality of fresh rice noodles [7,8,9,10]. In terms of raw material characteristics, previous studies have conducted extensive research on the relationship between rice quality indicators and fresh rice noodles. Relevant research suggests that the amylose content, gel consistency, and gelatinization temperature are the main rice quality indicators affecting the quality of fresh rice noodles [11]. Xuan et al.’s research indicated that the amylose content of rice between 20% and 25% can be a core parameter for making fresh rice noodles [12]. There is also research showing that the quality of fresh rice noodles is mainly related to the amylose content and gel consistency, with protein and lipids having little impact—rice with an amylose content of 22~27% and a gel consistency of 30~45 mm can produce better-quality rice noodles [3]. Therefore, there is still debate about the optimal values of raw material parameters suitable for making fresh rice noodles, and we speculate that this may be related to the length of the paddy storage period.

Proper aging of paddy can improve the quality of fresh rice noodles to a certain extent, and in production, paddy stored for one to three years is usually used for processing into fresh rice noodles [13]. This is mainly because fresh rice noodles made from paddy with a longer storage period have better tensile properties, shear resistance, chewiness, and elasticity [14]. However, a large number of studies have shown that paddy undergoes aging during storage, leading to changes in rice quality, with starch, fat, and protein in rice all undergoing changes [15], causing rice to lose its original color, aroma, taste, and nutritional components, as well as a decline in food quality [16], and even the production of toxic and harmful substances, such as aflatoxins [17], thus bringing potential risks to the processing of fresh rice noodles.

In response to this, this study is based on 14 types of paddies, harvested in the same period, and fresh rice noodles are made from 3 shorter paddy storage periods (6 months, 9 months, and 12 months). By comprehensively evaluating the differences in cooking quality, texture quality, and sensory scores of fresh rice noodles, the impact of the paddy storage period on the quality of fresh rice noodles is clarified, and the optimal storage period is determined. Furthermore, by analyzing the rice quality of the raw materials, the reasons for the differences in the quality of fresh rice noodles are analyzed. The aim is to provide a theoretical basis for the selection of the best storage period for fresh rice noodles’ raw materials, and at the same time, to offer new ideas for the optimization of safe processing of fresh rice noodles.

## 2. Materials and Methods

### 2.1. Experimental Materials

The test materials were 14 hybrid rice varieties: C-Liangyou 343 (V1), Zhongliangyou 3416 (V2), Xiangliangyou 2 (V3), Shenliangyou 5183 (V4), Chuanxiangyou 1101 (V5), Fengliangyou 373 (V6), Liangyou 336 (V7), Liangyou 5836 (V8), T-You 817 (V9), Huailiangyou 608 (V10), Longliangyou 760 (V11), Zhongliangyou 3418 (V12), Longliangyou 018 (V13), and Liang You 121 (V14). All seeds were provided by the Rice Research Institute of Hunan Agricultural University.

### 2.2. Experimental Design

Field experiments were conducted in Yanxi (28°30′ N, 113°83′ E), Hunan Province, China, from April to September in 2018. The experimental site is located in the central Hunan region and has a humid mid-subtropical monsoon climate. The average annual temperature is 16.7~18.2 °C, the average annual rainfall is 1457~2247 mm, and the sunshine duration is 1490~1850 h. The soil of the experimental field was a clay with medium fertility.

The cultivars were arranged in a completely randomized block design with three replications and a plot size of 40 m^2^. Pre-germinated seeds were sown in seedbeds on 25 April. Seedlings were manually transplanted on 24 May. Transplanting was performed at a hill spacing of 20.0 cm by 20.0 cm, with two seedlings per hill. In the experiment, 150 kg N hm^−2^, 75 kg P_2_O_5_ hm^−2^, and 75 kg K_2_O hm^−2^ were applied. Nitrogen fertilizer was applied at the proportion of 40% base fertilizer, 30% tillering fertilizer, and 30% panicle fertilizer. Phosphate fertilizer was used as the base fertilizer. Potassium fertilizer was applied at the ratio of 50% base fertilizer and 50% panicle fertilizer. Rice plants were grown according to local standard practices. In addition, the experiment was designed with three storage treatments, where the harvested seeds were stored for 6 months, 9 months, and 12 months, respectively, denoted as T6M, T9M, and T12M treatments.

### 2.3. Determination Items and Methods

#### 2.3.1. Rice Quality

Processing quality determination: Paddy was milled on a paddy mill (JLG-II, North China Experimental Instrument Co., Ltd., Tianjin, China) to obtain brown rice samples, and the brown rice rate was measured. Then, brown rice samples were milled into milled rice on a rice milling machine (JGMJ8098, Shanghai Jiading Grain and Oil Instrument Co., Ltd., Shanghai, China), and the milled rice rate was measured.

Appearance quality determination: An appropriate amount (about 15 g) of milled rice grains was used with a rice appearance quality determiner (SC-E, Hangzhou Wan Sheng Inspection Co., Ltd., Hangzhou, China) to measure the length-to-width ratio, transparency, and head rice yield, with each treatment repeated three times.

Amylose content: We accurately weighed 100 mg ± 1 mg of the rice flour sample and reference sample into a 100 mL volumetric flask, added 1.0 mL of anhydrous ethanol (Xi’an Sanpu Chemical Co., Ltd., Xi’an, China), and gently shook the volumetric flask to ensure the rice flour was well dispersed. Then, we added 9.0 mL of sodium hydroxide solution (Shanghai Runjie Science and Technology Development Co., Ltd., Shanghai, China) and shook the volumetric flask to mix the rice flour evenly. After that, it was placed in a boiling water bath for 10 min, then removed and cooled to room temperature, and we made up the volume with distilled water. Next, we took 5 mL of the sample solution into a 50 mL volumetric flask, added about 30 mL of distilled water, used a pipette to add 1 mL of 1.0 mol/L glacial acetic acid (Shanghai Aladdin Bio-Chem Technology Co., Ltd., Shanghai, China) into the volumetric flask, then added 1 mL of 0.2% iodine and potassium iodide solution into the volumetric flask, and made up the volume to 50 mL (timing was started immediately after adding iodine and potassium iodide (Zibo Wankang Pharmaceutical Chemical Co., Ltd., Zibo, China), and colorimetry was performed after 20 min).

Alkaline digestion value: We randomly selected 6 mature and plump whole milled rice grains and placed them in a square box, then added 10.0 mL of 1.70% potassium hydroxide (Huarong Chemistry Co., Ltd., Chengdu, China) solution. We used a glass rod to evenly distribute the rice grains and cover the box. Then, the box was transferred to a constant-temperature chamber set at 30 ± 2 °C and the temperature was maintained for about 24 h. After that, we removed the box and observed the decomposition of the endosperm of each rice grain one by one and recorded the grading according to the standard.

Gel consistency: We accurately weighed 100 mg ± 1 mg of the rice flour sample into a test tube, added 0.2 mL of 0.025% thymolphthalein blue ethanol (Tianjin Yongda Chemical Reagent Co., Ltd., Tianjin, China) solution, and gently shook the test tube or used a vortex mixer to ensure thorough dispersion of the rice flour. Then, we added 2.0 mL of 0.200 mol/L potassium hydroxide solution and shook the test tube to ensure the rice flour was well mixed. The test tube was immediately placed into a boiling water bath, the mouth of the test tube was covered with a glass marble, and it was heated in the boiling water bath for 8 min. We removed the test tube, took off the glass marble, let it stand and cool for 5 min, then placed the test tube in an ice water bath at around 0 °C for 20 min. Next, we removed the test tube from the ice water bath, placed it immediately on a leveled platform, such as a rice glue length measuring box or an incubator with a marked scale that was previously adjusted to be level, aligning the bottom of the test tube with the marked starting line, and let it stand for 1 h at a temperature of 25 ± 2 °C. After this period, we immediately measured the length of the rice glue flow within the test tube.

#### 2.3.2. Preparation of Fresh Rice Noodles

We soaked the rice material in water at 30 °C for 3 h, then ground it into a paste with a rice-to-water mass ratio of 1:1.8. The paste was passed through a sieve (aperture size 0.25 mm) and mixed evenly. We weighed out 60 g of rice paste and spread it evenly on a stainless-steel round plate, placed it in a steamer over boiling water, steamed it for 90 s, removed it, let it cool at room temperature for 15 min, then aged it at 4 °C for 2 h. After aging, we cut the paste into noodles that were 8 mm wide and 20 cm long and packed them in self-sealing bags for later use.

#### 2.3.3. Determination of Cooking Quality of Fresh Rice Noodles

(1) Broken noodle rate determination: We referred to the method of Luo et al. [18] to determine the broken noodle rate of the fresh rice noodles. We selected 20 pieces of fresh rice noodles that were 20 cm long, boiled them in 500 mL of boiling water for 1 min, then lifted the noodle samples and rinsed them in cold water to drain. The number of noodle pieces longer than 10 cm (X_1_) was recorded, and we calculated the broken noodle rate using the following formula:

Broken noodle rate/%= (20 − X_1_)/20 × 100% (1)

In the formula, X_1_ is the number of noodle pieces longer than 10 cm.

(2) Spit pulp value determination: Following the method of Cham et al. [19], we determined the spit pulp value of fresh wet rice noodles by measuring the moisture content (W). We weighed approximately 20 g of the rice noodle sample (M_0_), cooked it in 500 mL of boiling water for 2 min, then adjusted the volume of the broth to 500 mL. We transferred 50 mL of the broth to a vessel that was pre-weighed to a constant mass (M_1_), then dried it at a temperature of 105 ± 2 °C until it reached a constant mass (M_2_). The spit pulp value was calculated using the following formula:

Spit pulp value/%= [10 × (M_2_ − M_1_)]/[M_0_ × (1 − W)] × 100% (2)

In the formula, M_0_ represents the mass of the rice noodle sample, M_1_ represents the mass of the vessel that was weighed to a constant mass, M_2_ represents the combined mass of the vessel and the solid residue from the transferred broth, and W represents the moisture content of the rice noodles.

#### 2.3.4. Texture Determination of Fresh Wet Rice Noodles

The texture profile of fresh and wet rice noodles was determined using a texture analyzer (TA-XT2i Plus, Stable Micro Systems, Godalming, UK), with specific settings for the texture analyzer, referring to the method of Cham et al. [19]. The parameters for hardness, adhesiveness, elasticity, and chewiness were measured.

#### 2.3.5. Sensory Evaluation of Fresh Rice Noodles

Sensory evaluation is a scientific method used to measure, analyze, and interpret the reactions of products through sight, smell, touch, taste, and hearing. Sensory evaluation is a traditional method to evaluate the quality of rice and wheat flour food products. It is used because the indicators measured by chemical methods cannot accurately explain the overall situation and the interaction of various sensory elements. Therefore, the sensory evaluation plays an indispensable role in the food industry. In the process of sensory evaluation, sensory panels are requested to provide a note on the range of intensity in scale, which can later be converted into a score ranging between 0 and 100. In this study, the approach of sensory evaluation refers to Li’s method [20].

A sensory evaluation group consisting of 7 people rated the fresh rice noodles in a concentrated period, as shown in Table 1 [20]. The 7 evaluators had professional background and professional training. They evaluated the noodles in strict accordance with the sensory evaluation scoring rules. The results excluded the highest and lowest scores, and the average value was taken. The main purpose of removing the maximum score and the minimum score in the scoring was to eliminate the influence of extreme values caused by personal preferences on the average score, to make the sensory evaluation scoring more equitable. 

#### 2.3.6. Statistical Analysis

The data were entered using Microsoft Excel 2016 and analyzed using SPSS 25.0 software for one-way analysis of variance (ANOVA). Mean variances were separated using Duncan’s multiple range test, with the level of significant difference indicated as *p* < 0.05. The grey relational analysis was conducted according to the method of Hua et al. [21]. Principal component analysis and membership function analysis were performed following the methods of Yang et al. [22].

## 3. Results

### 3.1. Comparison of the Cooking Quality of Fresh Rice Noodles After Different Rice Storage Periods

**Figure 1:**
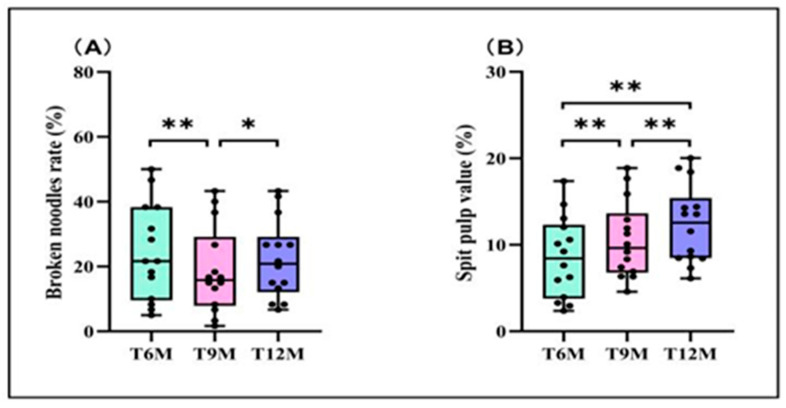
Effects of different rice storage periods on cooking quality of fresh wet rice noodles. (**A**) Broken noodle rate and (**B**) spit pulp value. Data are the mean (*n* = 14). *, Significant at the 0.05 probability level (*p* < 0.05); **, significant at the 0.01 probability level (*p* < 0.01).

The broken noodle rate of fresh rice noodles exhibited a trend of initially decreasing and then increasing with the extension of the rice storage period (Figure 1A), with the broken noodle rates for treatments T6M and T12M significantly increasing by 30.57% and 18.47% compared to the T9M treatment, respectively. The spit pulp value of fresh rice noodles showed an increasing trend with the extension of the rice storage period (Figure 1B). The spit pulp value for the T12M treatment significantly increased by 30.94% and 14.60% compared to the T6M and T9M treatments, respectively.

### 3.2. Comparison of the Cooking Indicators of Fresh Rice Noodles After Different Rice Storage Periods

**Figure 2:**
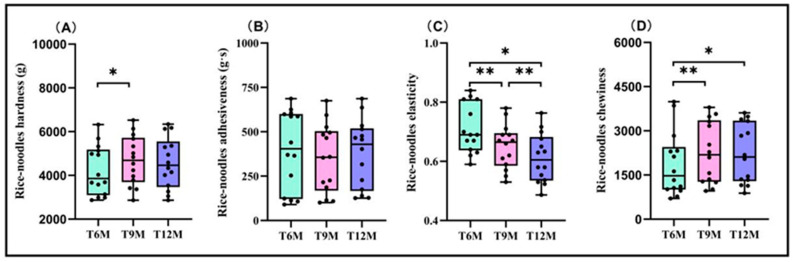
Effects of different rice storage periods on the texture index of fresh wet rice noodles. (**A**) Rice noodles’ hardness, (**B**) rice noodles’ adhesiveness, (**C**) rice noodles’ elasticity, and (**D**) rice noodles’ chewiness. Data are the mean (*n* = 14). *, Significant at the 0.05 probability level (*p* < 0.05); **, significant at the 0.01 probability level (*p* < 0.01).

The hardness and chewiness of fresh rice noodles showed a trend of initially increasing and then decreasing with the extension of the rice storage period (Figure 2A,D). The hardness of the T9M treatment significantly increased by 9.50% compared to the T6M treatment, while the chewiness of the T9M and T12M treatments significantly increased by 17.89% and 16.41% compared to the T6M treatment, respectively. There were significant differences in the elasticity of the fresh rice noodles made under the three different rice storage periods (Figure 2C), and the trend was gradually decreasing. The T6M treatment increased by 8.45% and 13.89% compared to the T9M and T12M treatments, respectively. The adhesiveness of fresh rice noodles did not change with the rice storage period (Figure 2B).

### 3.3. Comparison of Sensory Scores of Fresh Rice Noodles After Different Rice Storage Periods

**Figure 3:**
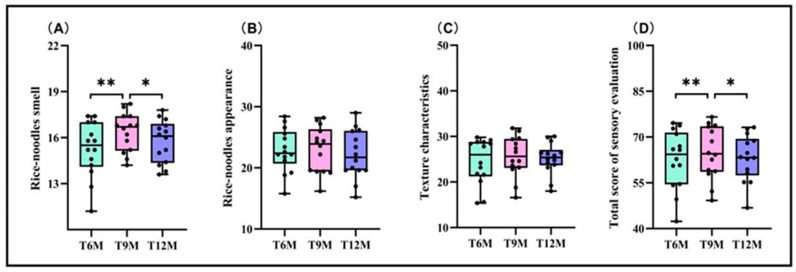
Effects of different rice storage periods on sensory scores of fresh wet rice noodles. (**A**) Rice noodles’ smell, (**B**) rice noodles’ appearance, (**C**) texture characteristics, and (**D**) total score of sensory evaluation. Data are the mean (*n* = 14). *, Significant at the 0.05 probability level (*p* < 0.05); **, significant at the 0.01 probability level (*p* < 0.01).

The appearance and texture characteristics of fresh rice noodles showed no significant differences between different storage periods (Figure 3B,C). There were significant differences in the smell scores and sensory total scores of the fresh rice noodles made after different rice storage periods, and both of these indicators showed a trend of increasing and then decreasing with the extension of the rice storage period (Figure 3A,D). The rice noodles’ smell scores of the T9M treatment increased by 6.72% compared to the T6M treatment and by 3.67% compared to the T12M treatment, while the sensory total score of the T9M treatment increased by 3.50% compared to the T6M treatment and by 2.75% compared to the T12M treatment.

### 3.4. Membership Function Analysis of Fresh Rice Noodle Quality

**Figure 4:**
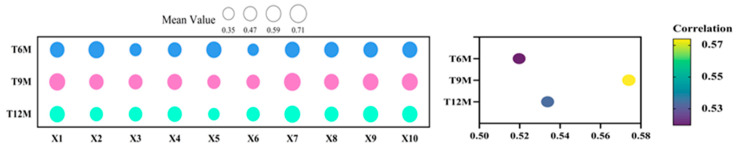
Membership function analysis of fresh wet rice noodles’ quality under different storage periods. X1: broken noodle rate; X2: spit pulp value; X3: rice noodles’ hardness; X4: rice noodles’ adhesiveness; X5: rice noodles’ elasticity; X6: rice noodles’ chewiness; X7: rice noodles’ smell; X8: rice noodles’ appearance; X9: texture characteristics; X10: total score of sensory evaluation.

The analysis of the impact of different rice storage periods on the quality of fresh rice noodles using the membership function method showed that the mean membership function values for treatments T6M, T9M, and T12M were 0.53, 0.57, and 0.52, respectively (Figure 4). The T9M treatment produced fresh rice noodles with the highest mean membership function value, indicating that among these three rice storage periods, the T9M period was the most suitable for processing into fresh rice noodles.

**Figure 5:**
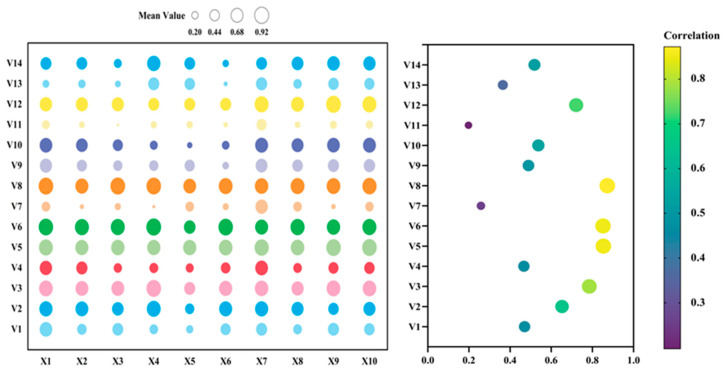
Membership function analysis of fresh wet rice noodles’ quality under different rice varieties. X1: broken noodle rate; X2: spit pulp value; X3: rice noodles’ hardness; X4: rice noodles’ adhesiveness; X5: rice noodles’ elasticity; X6: rice noodles’ chewiness; X7: rice noodles’ smell; X8: rice noodles’ appearance; X9: texture characteristics; X10: total score of sensory evaluation.

Further analysis using the membership function method on the fresh rice noodles made from 14 rice varieties under the T9M treatment (Figure 5) revealed that among the 14 varieties tested under the T9M treatment, those made from V5, V6, and V8 as raw materials had better quality, with membership function means of 0.85, 0.85, and 0.87, respectively. In contrast, the quality of fresh rice noodles made from V7, V11, and V13 was poorer, with membership function means of 0.26, 0.20, and 0.36, respectively.

### 3.5. Comparison of Rice Quality Among Different Rice Varieties

**Figure 6:**
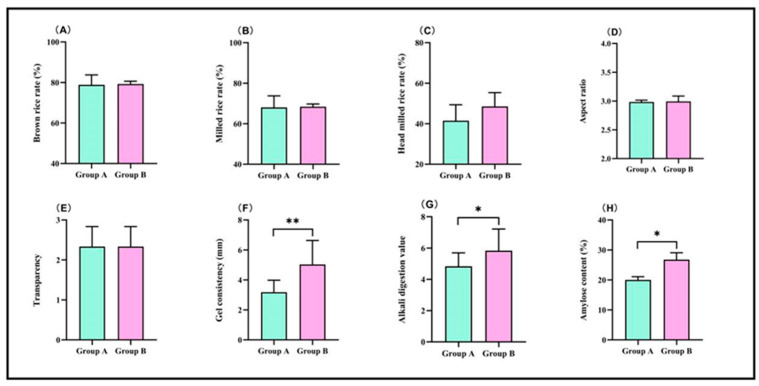
Comparison of rice quality among different rice varieties. (**A**) Broken rice rate, (**B**) milled rice rate, (**C**) head milled rice rate, (**D**) aspect ratio, (**E**) transparency, (**F**) gel consistency, (**G**) alkali digestion value, and (**H**) amylose content. Group A: V5, V6, and V8; Group B: V7, V11, and V13. *, Significant at the 0.05 probability level (*p* < 0.05); **, significant at the 0.01 probability level (*p* < 0.01).

To clarify the reasons for the differences in quality of the fresh rice noodles made from different rice varieties stored for nine months, we categorized the three varieties that produced better-quality fresh rice noodles (V5, V6, and V8) into Group A and the three varieties that produced poorer-quality fresh rice noodles (V7, V11, and V13) into Group B, and then further measured the rice quality of Group A and Group B. Through the analysis of the rice quality of Group A and Group B (Figure 6), the results indicated that there were no significant differences in the various indicators of processing quality and appearance quality between Group A and Group B, while significant differences were observed in cooking quality and nutritional quality. Specifically, the gel consistency, alkali digestion value, and amylose content for Group A were 32 mm, 4.8, and 20.02%, respectively; for Group B, they were 50 mm, 5.8, and 26.75%, respectively. Group A’s gel consistency, alkali digestion value, and amylose content were significantly lower than those of Group B by 36.00%, 17.24%, and 25.16%, respectively.

### 3.6. Correlation Analysis Between Rice Quality and Fresh Rice Noodle Quality

**Figure 7:**
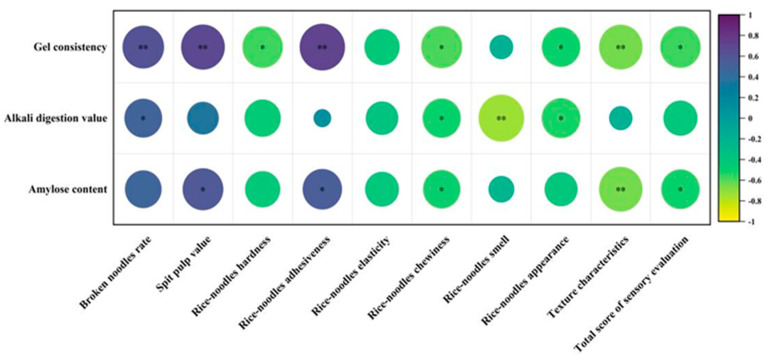
Correlation analysis between rice quality and fresh rice noodles’ quality (V5, V6, and V8). *, Significant at the 0.05 probability level (*p* < 0.05); **, significant at the 0.01 probability level (*p* < 0.01).

The analysis of the differences in rice quality between Group A and Group B identified significant differences in the gel consistency, alkali digestion value, and amylose content, but the differences among the data were not unique, and the relationships between the indicators were complex. To further reveal the relationship between rice quality and the quality of fresh rice noodles, a correlation analysis was conducted between the gel consistency, alkali digestion value, and amylose content of the varieties in Group A and Group B treated with T9M and various indicators of fresh rice noodles (Figure 7). The results showed that gel consistency was extremely significantly positively correlated with the broken noodle rate, spit pulp value, and adhesiveness, while it was significantly or extremely significantly negatively correlated with hardness, chewiness, appearance texture characteristics, and sensory total score. The alkali digestion value was significantly positively correlated with the broken noodle rate, while it was significantly or extremely significantly negatively correlated with chewiness, aroma, and appearance. Amylose content was significantly positively correlated with the spit pulp value and adhesiveness, and significantly or extremely significantly negatively correlated with chewiness, texture characteristics, and sensory total score.

### 3.7. Principal Component Analysis of Different Rice Varieties

**Figure 8:**
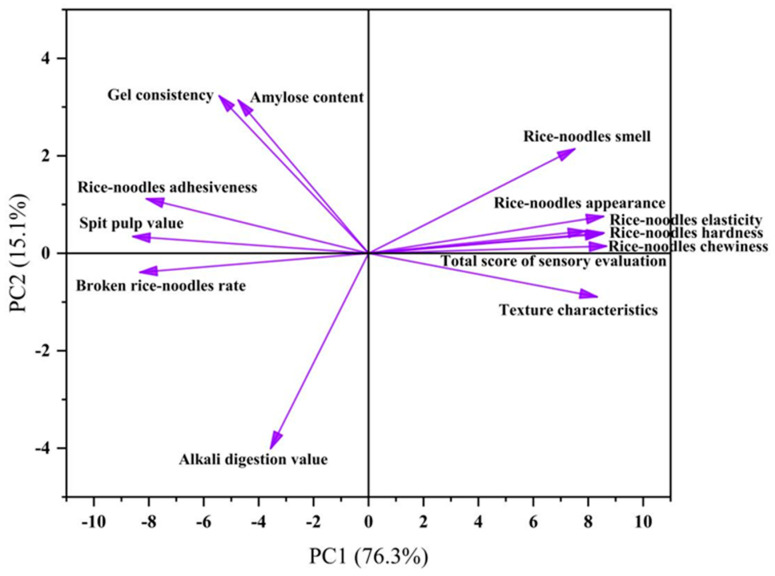
Principal component analysis of different rice varieties.

The complex correlations between the gel consistency, alkali digestion value, and amylose content with multiple indicators of fresh rice noodles suggest that there is different information overlap and intersection among the indicators, and it is not feasible to use a single or a few indicators directly as the main factors affecting the quality of rice varieties. Therefore, to comprehensively and systematically evaluate the quality of fresh rice noodles, we need to eliminate the shortcomings of evaluating rice quality with a single indicator. We employed the principal component analysis method to conduct an integrated evaluation of the quality of fresh rice noodles from two groups of varieties (Figure 8). The results showed that PC1 and PC2 accounted for 76.3% and 15.1% of the contribution rate, respectively, with a cumulative contribution rate of the two principal components reaching 91.4%. Thus, the two extracted principal components represented most of the information regarding the quality of rice and fresh rice noodles for Group A and Group B under the T9M treatment. The eigenvalue of PC1 was 9.917, with significant loadings on the sensory total score, hardness, broken noodle rate, and spit pulp value, with eigenvalues of 0.315, 0.312, −0.303, and −0.312, respectively. These four components mainly reflected the quality of fresh rice noodles; moreover, the eigenvalues of PC1 for the gel consistency, alkali digestion value, amylose content, broken noodle rate, spit pulp value, and adhesiveness were all negative. The eigenvalue of PC2 was 1.963, with significant loadings on the gel consistency, alkali digestion value, and amylose content, with eigenvalues of 0.485, −0.601, and 0.472, respectively. These three components were all indicators of rice quality.

### 3.8. Grey Relational Analysis Between Rice Quality and Fresh Rice Noodle Quality

**Figure 9:**
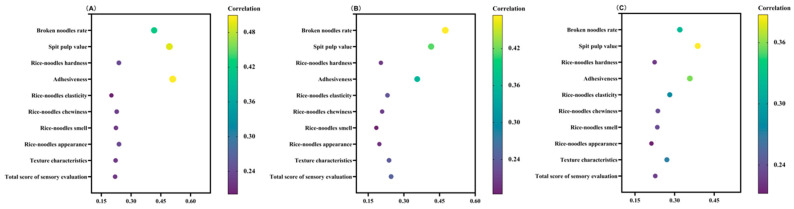
Grey correlation analysis between rice quality and the fresh rice noodles index. (**A**) Gel consistency, (**B**) alkali digestion value, and (**C**) amylose content.

To further analyze the relationship between the gel consistency, alkali digestion value, and amylose content with the quality of fresh rice noodles, a grey relational analysis was conducted between these parameters and various indicators of fresh rice noodles for the varieties in Group A and Group B treated with T9M (Figure 9). The grey relational coefficients between the gel consistency, alkali digestion value, and amylose content with various indicators of fresh rice noodles were as follows: 0.2006 to 0.5097, 0.1840 to 0.4740, and 0.2122 to 0.3875, respectively. This indicated that the grey relational coefficients between gel consistency and various quality indicators of fresh rice noodles were relatively high, confirming the results from the correlation analysis that showed a higher correlation between gel consistency and the quality indicators of fresh rice noodles. Additionally, the results indicated that the highest grey relational coefficients for the gel consistency, alkali digestion value, and amylose content with the quality indicators of fresh rice noodles were all for the broken noodle rate, spit pulp value, and adhesiveness, suggesting a high degree of index similarity between these indicators. This also confirmed the results from the principal component analysis, where the gel consistency, alkali digestion value, amylose content, broken noodle rate, spit pulp value, and adhesiveness all resided in the negative eigenvalues of PC1.

## 4. Discussion

This study analyzed the quality of fresh rice noodles made from rice grains stored for three different periods and found that the broken noodle rate in cooking quality showed a trend of decreasing and then increasing with the extension of the rice storage period. The reason for the decrease in the broken noodle rate may be related to the oxidation of protein molecules’ thiol groups to disulfide bonds, which leads to the cross-linking of low-molecular-weight proteins into high-molecular-weight proteins [23]. The reason for the subsequent increase in the broken noodle rate may be due to severe deterioration of rice quality, which fails to form an orderly gel structure, leading to easy breakage during cooking. Relevant studies have shown that the spit pulp value tends to gradually increase with the extension of the rice storage period [24], which is consistent with the results of this study. The reason for the gradual increase in the spit pulp value may be that microorganisms attached to the rice grains decompose starch after storage, leading to an increase in the spit pulp value [25]. In addition, we found that the changes in texture indicators also had a certain pattern. Hardness and chewiness showed a trend of increasing and then decreasing, adhesiveness showed a trend of increasing and then decreasing, and elasticity showed a gradual decreasing trend. The reason for the changes in the texture of fresh rice noodles may be related to the hydrolysis and oxidation of fats during the storage of rice grains, generating free fatty acids, which further combine with amylose to form starch-lipid compounds [26,27]. The results of sensory evaluation showed that the sensory total score of the T9M treatment was 3.50% and 2.75% higher than that of the T6M and T12M treatments, respectively.

To further analyze the quality differences of fresh rice noodles made from various treatments, we conducted a comprehensive scoring using the membership function analysis for the three treatments. The scoring results showed that the mean membership function value for the T9M treatment was 7.02% higher than that of the T6M treatment and 8.77% higher than that of the T12M treatment. From this, it can be observed that aging rice for a certain period can improve the quality of fresh rice noodles, but beyond a certain point in aging time, it can lead to a decline in the quality of fresh rice noodles. This finding is similar to the research results of Zhang et al. [28].

To further explore the reasons for the differences in quality of fresh rice noodles made from various treatments, we conducted a comprehensive scoring of the three treatments using the membership function analysis. The results indicated that the mean membership function value for the T9M treatment was 7.02% higher than that of the T6M treatment and 8.77% higher than that of the T12M treatment. This suggests that aging rice for a certain period can enhance the quality of fresh rice noodles, but beyond a certain point, the aging time leads to a decline in the quality of fresh rice noodles. This finding is similar to the research results of Zhang et al. [28]. To delve into the causes of quality differences in fresh rice noodles, we categorized 14 rice varieties into 2 groups based on the membership function analysis: Group A with the highest mean membership function values and Group B with the lowest. Analysis of the rice quality of these two groups revealed significant differences in quality indicators, such as the gel consistency, alkali digestion value, and amylose content. The varieties in Group A had a gel consistency of about 32 mm and an amylose content of about 20%, whereas the varieties in Group B had a gel consistency of about 50 mm and an amylose content of about 27%. Relevant studies suggest that rice with an amylose content of 20–25% [12] and a gel consistency of 30–45 mm [3] can be used as core indicators for selecting varieties suitable for rice noodle production, which is similar to the results of this study. Other studies also indicate that rice with an amylose content above 20% should be used for making rice noodles, and when the amylose content exceeds 26%, it leads to a decline in the quality of rice noodles [29]. These results support the rationality of this study’s approach to selecting Group A and Group B rice varieties for further comprehensive analysis using the membership function.

Next, we conducted a correlation analysis between the quality indicators of rice and fresh rice noodles for these two groups of rice varieties. The correlation results indicated that within a certain range, gel consistency was significantly positively correlated with the broken noodle rate, spit pulp value, and adhesiveness. An increase in gel consistency can decrease the quality of fresh rice noodles, which is consistent with relevant research findings [11]. To further clarify the roles of the gel consistency, alkali digestion value, and amylose content in the formation of fresh rice noodle quality, principal component analysis revealed that the gel consistency, alkali digestion value, amylose content, broken noodle rate, spit pulp value, and adhesiveness were negative characteristic indicators of PC1. This suggests that within the range of the gel consistency, alkali digestion value, and amylose content for Group A and Group B varieties, the quality of fresh rice noodles was mainly improved by affecting indicators such as the broken noodle rate, spit pulp value, and adhesiveness. Finally, to verify the results obtained from the principal component analysis, we conducted a grey relational analysis and found that the gel consistency, alkali digestion value, and amylose content all had high correlation coefficients with the broken noodle rate, spit pulp value, and adhesiveness among the quality indicators of fresh rice noodles. Moreover, the correlation coefficient of gel consistency with the quality indicators of fresh rice noodles was the highest, which is similar to relevant research findings [30,31].

In summary, the correlation coefficient between gel consistency and the quality indicators of fresh rice noodles was the highest, which was also supported by the results of the correlation analysis. This indicates that for a nine-month rice storage period, gel consistency was the most critical factor affecting the quality of fresh rice noodles. Within the range of 32–50 mm, an increase in gel consistency led to a decline in the quality of fresh rice noodles. The reason may be that the lower the gel consistency, the higher the rice’s holding viscosity, final viscosity, and setback value, and the better the gel performance, thereby resulting in better-quality fresh rice noodles. Although this study did not determine the paste properties of the rice flour, it is well known that the paste properties of rice flour are closely associated with its composition and fresh rice noodle quality [32]. A lower gel consistency will lead to a higher viscosity and pasting temperature [33].

In addition, this study only discussed the effect of storage periods of 6 months, 9 months, and 12 months on the fresh rice noodles’ quality. The effect of a storage period of more than one year on the fresh rice noodles’ quality is not clear. In this study, 14 varieties were prepared, and each variety may have varying responses to different storage periods. The response of different varieties to the change in the storage period needs further study.

## 5. Conclusions

Rice grains stored for nine months yielded the best-quality fresh rice noodles when processed, characterized by a low broken noodle rate, low spit pulp value, high hardness, low adhesiveness, high elasticity, high chewiness, and high sensory total score. This study indicated that the key factor affecting the quality of fresh rice noodles during a nine-month rice storage period was the gel consistency of the rice grains. When the gel consistency of the rice grains was around 32 mm, the quality of the fresh rice noodles was superior. Therefore, selecting rice varieties with a gel consistency of around 32 mm after nine months of storage for the processing of fresh rice noodles could provide a new direction and strategy for the selection of raw materials for fresh rice noodles.

## Figures and Tables

**Table 1:** Fresh rice noodles sensory evaluation scoring rules.

Level 1 Indicators/Scores	Secondary Indicators/Scores	Specific Characteristic Description: Score
Smell/25 points	Rice aroma/25 points	With the rice aroma, there is a rich aroma: 22–25 points
		With the rice aroma, the aroma is not obvious: 18–21 points
		No rice aroma, but no peculiar smell: 15–17 points
		No rice aroma, and peculiar smell: 0–14 points
Appearance/35 points	Color/10 points	Color is white: 8–10 points
		Color is normal: 5–7 points
		Color is yellow or gray: 0–4 points
	Gloss/10 points	Obvious gloss: 8–10 points
		Slightly shiny: 5–7 points
		Matte: 0–4 points
	Structure/15 points	The structure is tight, the chopsticks do not easily break the strip, there is no merging of strips,
		crushing, or cracking: 10–15 points
		No broken noodles, parallel strips, crushed powder, a small amount of cracking: 5–9 points There is crushed powder, it is easy to break the noodles or have a parallel strip, cracking: 0–4 points
Texture characteristics/40 points	Adhesiveness/10 points	Smooth and non-sticky: 8–10 points
		Basic non-sticky teeth: 5–7 points
		Sticky teeth: 0–4 points
	Rice noodle hardness/10 points	Moderate hardness: 8–10 points
		Slightly hard or soft: 5–7 points
		Very soft or very hard: 0–4 points
	Sense of strength/10 points	Chewy: 8–10 points
		Slightly chewy: 5–7 points
		Non-chewy: 0–4 points
	Rice noodle elasticity/10 points	Elasticity: 8–10 points
		Elasticity is average: 5–7 points
		Insufficient elasticity: 0–4 points

## Data Availability

The original contributions presented in the study are included in the article. Further inquiries can be directed to the corresponding author.
